# Pacific Islands Cohort on Cardiometabolic Health Study: rationale and design

**DOI:** 10.1186/s12889-022-13783-9

**Published:** 2022-07-27

**Authors:** Rachael T. Leon Guerrero, Margaret P. Hattori-Uchima, Grazyna Badowski, Tanisha F. Aflague, Kathryn Wood, Kristi Hammond, Remedios Perez

**Affiliations:** University of Guam, UOG Station, 303 University Drive, Mangilao, Guam 96923

**Keywords:** Child obesity, Guam, Palau, Pohnpei, Micronesia, Cardiometabolic, Metabolic syndrome, Non-communicable diseases

## Abstract

**Background:**

Pacific Islanders, including those residing in the US Affiliated Pacific Islands (USAPI), experience some of the highest mortality rates resulting from non-communicable diseases (NCDs) worldwide. The Pacific Island Health Officers’ Association declared a Regional State of Health Emergency in 2010 due to the epidemic of NCDs in the USAPI. Obesity, a known risk factor for NCDs, has become an epidemic among both children and adults in Micronesia and other parts of the USAPI. There is some recent information about overweight and obesity (OWOB) among young children in the USAPI, but there is no data looking at the relationship between children and their biological parents. The Pacific Islands Cohort on Cardiometabolic Health (PICCAH) Study aims to collect data on NCD lifestyle factors from two generations of families (n = 600 child-parent dyads or 1,200 participants) living in Guam, Pohnpei, and Palau.

**Methods:**

The PICCAH Study is an epidemiological study using community-based convenience sampling to recruit participants in USAPI of Guam, Palau, and Pohnpei. The goal is to recruit participant dyads consisting of 1 child plus their biological parent in Guam (500 dyads or 1,000 participants), Pohnpei (50 dyads or 100 participants), and Palau (50 dyads or 100 participants). All participants are having the following information collected: demographic, health, and lifestyle information; anthropometry; diet; physical activity; sleep; acanthosis nigricans; blood pressure; and serum levels of fasting plasma glucose, fasting insulin, glycated hemoglobin, total cholesterol, triglycerides, LDL, and HDL.

**Discussion:**

The PICCAH Study is designed to establish the baseline of a generational epidemiologic cohort with an emphasis on cardiometabolic risk, and to better understand the extent of DM and CVD conditions and related risk factors of those living in the USAPI jurisdictions of Guam, Pohnpei, and Palau. This study also serves to further build research capacity in the underserved USAPI Region.

**Supplementary Information:**

The online version contains supplementary material available at 10.1186/s12889-022-13783-9.

## Background

Non-communicable diseases (NCDs) such as cardiovascular disease, cancer, respiratory diseases, and diabetes are a significant health problem worldwide. Each year, NCDs kill 41 million people worldwide, which is equivalent to 71% of all deaths [1, 2]. Factors related to overweight and obesity (OWOB) such as sleep, physical activity, psychosocial, life course exposure, socioeconomic status (SES), and diet [1–4] contribute to the incidence of NCDs. Pacific Islanders experience some of the highest mortality rates resulting from NCDs [2]; and the Pacific Island Health Officers’ Association (PIHOA) recognized a state of emergency for the United States Affiliated Pacific Island (USAPI) region in 2010, and through their Board Resolution #48–01 [5] declared a Regional State of Health Emergency due to the epidemic of NCDs in the USAPI.

Pacific Islanders residing in the USAPI region, which includes Hawaii, American Samoa, Guam, the Commonwealth of the Northern Mariana Islands, the Republic of the Marshall Islands, the Republic of Palau, and the Federated states of Micronesia (Chuuk, Yap, Pohnpei, and Kosrae), suffer from the dual burden of communicable diseases and NCDs [5, 6]. These culturally diverse groups of people in the USAPI represent more than 20 ethnic subgroups with indigenous linkages across the Pacific regions of Micronesia, Polynesia, and Melanesia [6]. Guam is part of Micronesia, and is a U.S. Territory. CHamorus are the original inhabitants of Guam [7, 8], and are typically grouped with other Pacific Islanders in national surveys. The current population of Guam is characterized by substantial ethnic variation [9]: 37% CHamoru, 26% Filipino, 12% other Pacific Islander, 11% other ethnicity, 7% White, and 7% other Asian. There is less ethnic diversity in the other Pacific Island region of Palau, with the majority (73%) of Palau’s population being Palauan [10] (original inhabitants of Palau). Pohnpei is part of the Federated States of Micronesia (FSM) and Pohnpeians (original inhabitants of Pohnpei) make up 29.8% of the total population of FSM [11], but the specific ethnic breakdown of the population of Pohnpei is unknown. As with ethnicity, there are varied levels of development across the region. Guam is the most developed, with the other jurisdictions at varied levels as developing countries. Pacific Islanders are under-represented in health research, and most of the available research available aggregates information from both Pacific Islanders and Asian Americans, making it difficult to distinguish the actual disparities between and within these two groups [12].

OWOB is a known risk factor for NCDs [4, 13], and OWOB has become a major health problem in Micronesia [14]. OWOB among children has become a global epidemic affecting many countries [14–18], including the US and the USAPI. Children suffering from OWOB are at high risk for NCDs such as type 2 diabetes, cardiovascular diseases, negative health consequences such as respiratory diseases, other musculoskeletal complications, poor health-related quality of life, and negative emotional health [15, 16]; and OWOB during childhood tracks into adolescence and adulthood [17–19]. In the US, a high prevalence of OWOB among racial/ethnic minority groups for children 2–19 years is reported, specifically for Non-Hispanic black (19.5%) and Hispanic (21.9%) children, yet Native Hawaiian or Other Pacific Islanders are not included [20, 21]. We recently reported that the rate of OWOB in a sample of 865 children living on Guam was 27.4% [22], which was consistent with previous reports [21] and similar to the prevalence of OWOB in the US [23]; however, the majority of Guam children surveyed, even those not suffering from OWOB, did not meet recommendations for sedentary screen-time (83.11%), sleep duration (59.6%), fruit intake (58.7%) and vegetable intake (99.1%) intake, and sugar sweetened beverage (SSB) intake(73.7%) [13]. Although the rate of OWOB among children on Guam may be similar to rates in the US, studies show that children who suffer from OWOB have a higher risk of developing OWOB as adults [5] with an increased risk of chronic diseases later in life [15]. Given the poor health choices identified in our recent study [22], children on Guam are in danger for developing NCDs later in life as evidenced by the high prevalence of adult NCDs [24].

Despite the recent information about OWOB among young children in Guam [22], there is no data looking at the relationship between children and their biological parents. Therefore, the Pacific Islands Cohort on Cardiometabolic Health (PICCAH) Study aims to collect data on NCD lifestyle factors from two generations of families (*n *= 600 child-parent dyads or 1,200 participants) living in Guam, Pohnpei, and Palau. The purpose of this paper is to present the design and rationale of the PICCAH Study being conducted in Guam, Pohnpei, and Palau.

## Methods/design

The PICCAH Study is the first epidemiological study conducted on Guam, Pohnpei and Palau to research NCD risk factors among children and their biological parents. Specifically, the purpose of PICCAH was to estimate the prevalence of diabetes mellitus (DM) and cardiovascular disease (CVD), as well as the presence of lifestyle factors (acculturation, diet, physical activity, sleep patterns, stress levels, blood pressure, body size, and tobacco, alcohol, and areca (betel) nut consumption) that contribute to NCDs among children and adult Pacific Islanders living in Guam, Pohnpei, and Palau, and to assess the relationship of those risk factors between children and their biological parents. Data gathered from this study will establish the baseline of a generational epidemiologic cohort with an emphasis on cardiometabolic risk on Guam, a pilot study in both Pohnpei and Palau, and to better understand the extent of DM and CVD conditions and related risk factors of those living in the USAPI jurisdictions of Guam, Pohnpei, and Palau. An innovative aspect of the PICCAH study is the recruitment of ‘dyads’, consisting of both young children, ages 4–10 years, and their biological parent. The goal is to recruit at least 500 evaluable child-parent dyads in Guam (*n* = 500 children and *n* = 500 adults), and 50 child-parent dyads on both Pohnpei (*n* = 50 children and *n* = 50 adults) and Palau (*n* = 50 children and *n* = 50 adults).

### Human subjects approval

The PICCAH Study is funded by the US National Institute of Minority Health Disparities (NIMHD). Ethical approval for this project was granted by the University of Guam Committee on Human Research Subjects (CHRS/IRB#19–171), the Institutional Review Board of the College of Micronesia-FSM (#20,171,803–20,190,802), and the Palau Institutional Review Board (#PIRB-2020–01). Written consent is given by all parents and children 7 years and older, and oral assent is given by all child participants prior to their inclusion, in accordance with the Declaration of Helsinki. In addition, approvals for working with teachers and parents of Guam Department of Education and Guam Head Start (a federally funded program that educates preschool-age children and their families) were received in coordination with the program directors and school principals when appropriate. Other local-level agreements in Guam, Pohnpei, and Palau include approvals from the chiefs, pastors, and mayors in participating villages.

### Power and sample size calculations

The adequacy of our sample size to detect differences between groups was based on the z-test comparing independent proportions [25], using α = 0.05 (two-sided) and β = 0.2. Our sample size was sufficient to detect small differences in prevalence (0.6–0.11) between generations of children and parents (*n* = 500 per group) and moderate differences (0.09–0.18) between subgroups within generation (children subgroup and parent subgroup) defined by sex (*n* = 250 per group) and by ethnic group (*n* = 166 per group). Larger differences (0.18–0.32) are detectable within generation between sex-ethnic subgroups (*n* = 83 per group) and between generations in Pohnpei (*n* = 50 per group) and Palau (*n *= 50 per group). For a sample sizes of 500, the range of the coefficient of variation % for a prevalence between 0.05–0.50 is 5–20%. In addition, the sample size has good power to test the ICC ≥ 0.5. With α = 0.05 (2-sided) and β = 0.20 and sample size of 500, the range of intraclass correlation coefficients that is detectable as significant is ≥ 0.58.

### Original participant recruitment plan for Guam — randomized sampling

To establish an epidemiological cohort of young children, ages 2–10 years, the initial recruitment plan was to use the Guam Early Hearing Detection & Intervention (EHDI) Network, which is housed at the University of Guam Center for Excellence for Developmental Disabilities Education and Research Services (UOG-CEDDERS). The Guam EHDI Network is a data system that collects information on all infants born on Guam to ensure all babies born on Guam have their hearing screening prior to hospital discharge, and receive follow-up screenings, diagnostic audiological evaluations, and enrollment in early intervention services if needed. The EHDI sampling frame was stratified by sex, ethnicity (CHamoru, Filipino, Other Micronesians) and birth year for a total of six strata. The goal was to recruit 90 children from each of the strata; and then children were to be randomized to recruitment of mother or father, to ensure that a balance of male and female parents was attained. The stratified sampling was to ensure that reasonably precise comparisons could be made between groups. Representative statistics for this Guam birth cohort was estimated by weighting to the overall EHDI population. There are over 42,000 individualized records of children born in Guam since November 2002. The records contain parent contact information and basic birth information about the children. The characteristics of children born in Guam between 2009 and 2014 are shown in Table 1.

**Table 1:** Birth characteristics (frequency or mean) of children in the Guam Early Hearing Detection & Intervention (EHDI) Network Database, 2009 to 2014

	**CHamoru**	**Chuukese**	**Filipino**	**Pohnpeian**	**Other Pacific Islander**	**Other**
Total # Children	6,079	3,233	2,479	347	498	2,228
Boys	51.4%	51.7%	51.8%	51%	50.8%	52.6%
Girls	48.6%	48.3%	48.2%	49%	49.2%	47.4%
Mean gestational age, wks	38.2	38.4	38.0	38.3	38.3	38.3
Mean weight, g	3,178.8	3,267.8	3,109.5	3,213.4	3,276.1	3,177.4

The phone number and address listed in the Guam EHDI database were used to contact parents; and a total of 14,864 letters of invitation were sent through US Postal Service to the addresses listed. In the official invitation letters, parents were informed about the PICCAH study, invited to participate in the research opportunity, and urged to call the research office if they were interested in the study. Out of the 14,864 letters sent out, only 4 dyads were successfully recruited. Research staff then attempted to contact parents from the Guam EHDI database via telephone, as a follow-up to the official letter of invitation. A total of 776 phone calls were made and of those, 56 parents said ‘yes’, but only 2 dyads were successfully recruited.

Despite efforts of the research staff during the first 20 months of the study, recruitment of participants was very low because using the Guam EHDI network to recruit and randomize participants did not work for this Pacific Islander community. As this study was considered a capacity-building project, and because of the extremely low response rate using the Guam EHDI network sampling frame, the federal sponsor (NIMHD) approved a change in recruitment protocol to non-randomized, non-stratified community-based participant recruitment.

### Revised participant recruitment plan — community-based convenience sampling

The research team decided to employ community-based, convenience sampling to recruit the remaining 496 dyads on Guam, as well as the 50 dyads on Pohnpei, and the 50 dyads on Palau. We implemented similar techniques on the 3 different Pacific Islands, with altered steps to address cultural and other specific factors within the communities in Guam, Pohnpei and Palau. This community-based, convenience sampling method included basic recruitment components such as: 1) establishing presence and trust through community outreach and awareness; 2) creating relationships with various groups, institutions, organizations, businesses, etc. with similar goals or similar target audiences for a wide-ranging approach; 3) partnering with multiple community champions for advocacy and coordination; 4) assessing logistics/basic infrastructure and planning standardized measurement/enrollment accordingly; and 5) obtaining basic knowledge of social and cultural etiquette for cultural approval and acceptance of research presence and data collection within the specific island location.

On Guam, the project staff use specific community-based techniques to increase participant recruitment such as: 1) Participating in local community outreach health events sponsored by institutions and businesses such as University of Guam, Guam Community College, Guam Department of Public Health & Social Services, Guam Department of Education, Guam Public Library, Guam Mayor’s Council, Guam Head Start, health insurance companies, and private medical clinics. Other community outreach events included community/village health fairs, summer camps, sports practices and games, and local 2 k/5 k running events; 2) Working with local churches and community centers to distribute informational flyers to members; 3) Contacting local elementary schools and Guam Head Start Program classrooms, first by attaining permission from school district superintendent and school principals, then providing letters of invitation to children (and their parents) who attended the school; and 4) Word of mouth—leveraging potential participants to recruitment known associates or family members. Potential participants who were interested in the study were asked to provide contact information so that project staff could contact them to schedule their interview.

On Pohnpei, recruitment sources were adjusted to create a strategy that involved community engagement with recruitment through partnerships with the College of Micronesia-FSM (COM), the Pohnpei Community Health Center (CHC), and local faith-based and social organizations. The Guam team was deployed to Pohnpei to conduct data collection, with additional community-based recruitment conducted using social, school-based, and church networks. Pohnpei-specific community-based recruitment strategies include: 1) partnering with trusted community health champions including the Pohnpei CHC; 2) going out to remote locations to collect data, rather than require participants to travel to COM; and 3) ensuring a native Pohnpeian speaker is present and actively assisting with data collection, which helps establish trust and ensures that participants could communicate in their native language.

On Palau, due to travel restrictions during the COVID-19 pandemic in 2020–2022, the study recruitment and data collection consists of online data collection. Recruitment and data collection is facilitated in coordination with the Palau Ministry of Health (MOH). Recruitment is primarily conducted through the partnership with the Ministry of Health, Palau Community College, word of mouth, and through the use of posters, radio, and news releases. Staff on Guam provide online assistance for data collection and are available to answer questions and provide assistance to participants. Community engagement is fostered through a local (Palauan) faculty from the Community College, and the MOH staff, who are key members of the Palauan health care system.

Before participant dyads (child and parent) are recruited into the study, a trained project staff reviews and determines their eligibility to participate in the study. Once the participant dyads are deemed eligible, they are asked to participate in 3 visits, are informed of the various surveys and measurements that will occur at each visit, and informed of the amount of remuneration for their participation in the study. Participant assessment measurements are listed in Table 2. Participant dyads in Guam are eligible if: 1) child was born on Guam between 2010 and 2014; 2) parent was between 18—50 years of age; 3) parent was biological parent of child. Participant dyads on Pohnpei are eligible if: 1) child is was born on Pohnpei between 2010 and 2014; 2) parent was between 18—50 years of age; 3) parent was biological parent or grandparent of child. Participant dyads in Palau are eligible if: 1) child was born on Palau between 2012 and 2018; 2) parent was between 18—50 years of age; 3) parent was biological parent or grandparent of child.

**Table 2:** Pacific Islands Cohort on Cardiometabolic Health (PICCAH) Program Assessment Measures of Child and Adult Participants

			**Guam**			**Pohnpei**		**Palau**	
**Category**	**Measurement**	**Measurement Tools**	**Visit 1**	**Visit 2**	**Visit 3**	**Visit 1**	**Visit 2**	**Visit 1**	**Visit 2**
***Child Measurements***									
Demographic	Demographic [26–31]	Questionnaire	X			X		X	
Stress	Stress [32]	Questionnaire	X			X		X	
Parent–Child PA Interaction	Parent–Child PA Interaction [33]	Questionnaire	X			X		X	
Physical Activity	6-day PA [34]	Accelerometer			X		X		X
Screen time	6-day ST [34]	Accelerometer			X		X		X
	Usual ST [35]	Questionnaire	X			X		X	
Sleep	Sleeping behavior [36]	Questionnaire	X			X		X	
	6-day sleeping [34]	Accelerometer			X		X		X
Diet	2-day food intake [37, 38]	2-Day Food Log			X				
	24-Hour Intake	24-Hour Recall				X		X	
Anthropometry	Height	Stadiometer	X			X		X	
	Weight	Portable scale	X			X		X	
	Waist circumference	Circumference tape	X			X		X	
Acanthosis Nigricans	Presence/Severity [39]	Visual Observation Assessment Form	X			X		X	
Blood Pressure	Blood Pressure	Sphygmomanometer	X			X		X	
Biospecimens	Cholesterol, Total			X			X		X
	Cholesterol, HDL			X			X		X
	Cholesterol, LDL			X			X		X
	Triglycerides			X			X		X
	Fasting Plasma Glucose			X			X		X
	Insulin			X					
	HA1c			X			X		X
***Adult (Parent) Measurements***									
Demographic	Demographic [26, 30, 31]	Questionnaire	X			X		X	
Medical History	Medical History [40]	Questionnaire	X			X		X	
Reproductive History	Reproductive History [40]	Questionnaire	X			X		X	
Culture	Culture [26–29]	Questionnaire	X			X		X	
Tobacco Use	BRFFS [31]	Questionnaire	X			X		X	
Alcohol Use	BRFFS [31]	Questionnaire	X			X		X	
Betel Nut Use	Guam BRFFS [31, 41]	Questionnaire	X			X		X	
Sleep	Daily Sleep (hrs/d)	Questionnaire	X			X		X	
Physical Activity	IPAQ [42]	Questionnaire	X			X		X	
Depression, Anxiety, Stress	DASS 21 [43]	Questionnaire	X			X		X	
Parent–Child PA Interaction	Parent–Child PA Interaction [44]	Questionnaire	X			X		X	
Diet	Usual diet intake [45]	Marianas FFQ			X				
	24-Hour Intake	24-Hour Recall				X		X	
Anthropometry	Height	Stadiometer	X			X		X	
	Weight	Portable scale	X			X		X	
	Waist circumference	Circumference tape	X			X		X	
Acanthosis Nigricans	Presence/Severity [39]	Visual Observation Assessment Form	X			X		X	
Blood Pressure	Blood Pressure	Sphygmomanometer	X			X		X	
Biospecimens	Cholesterol, Total			X			X		X
	Cholesterol, HDL			X			X		X
	Cholesterol, LDL			X			X		X
	Triglycerides			X			X		X
	Fasting Plasma Glucose			X			X		X
	Insulin			X					
	HA1c			X			X		X

If the participant agrees to be in the study, a project staff member reviews the consent form and waiver of liability with participants. After receiving signed consent by the participant, a copy of the consent form is given to the participant, while the original consent form and contact information is stored in a locked file separated from the rest of their de-identified data. Participants are also informed that they may withdraw from the study at any time without penalty. For the child participants over the age of 7 years, a project staff member obtains verbal and written assent from the child before any measurements are conducted.

Although most residents of Pohnpei and Palau speak English, we have a native Pohnpeian speaker and a native Palauan speaker as part of those respective data collection teams to ensure that participants could communicate in their native language, if needed, when participating in this study, answering questions, and undergoing measurements.

### Data collection

Visit 1, the initial interview, includes the review of the consent forms, administration of questionnaires, review of protocols on how to fill out the (child) 2-day food log, and the measurements (height, weight, waist circumference, blood pressure, and acanthosis nigricans neck photo). Parents are asked to complete the questionnaires for themselves (demographics, adult health and lifestyle questionnaire, and food intake) and their child (demographics and child health and lifestyle questionnaire). Dietary intake is assessed by either the Marianas Food Frequency Questionnaire (adults in Guam), 24-h dietary recall (adults and children on Pohnpei and Palau), or 2-day food logs (children in Guam). Physical activity is measured by accelerometers. Anthropometry (height, weight, waist circumference) and blood pressure measurements are collected on the same day. Visit 2 takes place at the health department laboratory for the blood collection. The project staff schedule the appointment with the laboratory and provided the participants with a written reminder, as well as a phone call reminder the day before. Visit 3 (only for Guam) takes place within two weeks of visit 1 to allow participants to return the food logs and accelerometers. Project staff call participants to remind and follow-up with visits. Table 2 provides a listing of all child and adult forms and assessments collected during the various visits by site.

### Demographic, health and lifestyle questionnaire

Adults are asked to complete a questionnaire about their child. This questionnaire assesses the demographic profile of the child including age, sex, race/ethnicity, birthplace, birth weight and length, education, household composition (adults and other children with their age, sex, and relationship to child), household food security, general health status, early life feeding behavior, screen-time, and sleep behaviors. Questions about stress and parent–child physical activity interactions arere both administered, via interview, directly to child participants, 7 years or older. All questions were adapted from those previously used in other studies conducted in this region with USAPI participants [26–33, 35, 46]. Adult participants are then asked to complete a questionnaire about themselves. The first questionnaire asks similar demographic questions about themselves such as: age, sex, date of birth, birthplace, number of years living on the island of residence (either Guam, Pohnpei, or Palau), race/ethnicity, education, marital status, religion, household food security, and general health status. Additional questions ask about medical/reproductive history, cultural identity and heritage, sleep, physical activity, depression and stress, parent–child interactions, betel (areca) nut use, tobacco use, and alcohol use. Questions were adapted from those previously used in other studies [26–29, 31, 36, 40–44].

### Anthropometry

For both children and parents, weight (kg), height (cm) and waist circumference (cm) are measured by trained research staff based on standardized procedures and protocols [47–49]. Zerfas criteria were used to standardize research staff against the height, weight, and waist measurement of a certified anthropometrist [50, 51]. Zerfas does not provide waist circumference criterion; however uniform criterion assigned to all assessments measured in cm (mm) units was used. Research staff could not assess anthropometry unless they passed the Zerfas criteria. Measurements are taken in a private setting. Participants are instructed to wear light clothing and asked to remove their shoes and hair ornaments and empty their pockets during measurements. Height is measured to the nearest 0.1 cm using portable stadiometers (Perspective Enterprises Stadiometer model PE-AIM-101). Weight is measured to the nearest 0.1 kg using portable scales (SECA scale Model 876; Chino, CA). Plastic tape (SECA 2001 measuring tape; Chino, CA) are used to measure waist circumference at the level of the umbilicus to the nearest 0.1 cm [47]. Height, weight, and waist circumference are measured three times, and three additional measures are made if there were no two measures among the original three within 2 units (e.g., 0.2 cm for height). These measures are used to compute BMI as weight (kg) / height (m)^2^, waist (cm) to height (cm) ratios for both children and adults. In addition, subsequent BMI z-score, waist circumference z-score, BMI-for-age-percentiles, and waist circumference-for-age percentiles are computed for children [52, 53]. Due to COVID-19 restrictions during data collection in Palau, self-reported height (in), weight (lg), and waist circumference (in) are being collected, with on-site verification by the Guam team within 1–2 months of data collection.

### Food frequency questionnaire (FFQ)

For adults recruited in Guam, self-reported dietary intake for the previous year is assessed from the validated Marianas FFQ [45], which was developed by researchers from the University of Guam and the University of Hawaii Cancer Center to assess nutrient intake for adults living in Guam and the Mariana Islands. Food lists for this FFQ were generated from 24-h recalls, and the final FFQ was modeled after the Multi-Ethnic Cohort (MEC) FFQ [40, 45]. Validity and reliability for the Marianas FFQ was established for both CHamoru and Filipino adults living in Guam and the other Mariana Islands [45]. Adult/parent participants are given the FFQ at visit 1 and research staff instructs the parent on how to complete the FFQ. Adults/parents complete the FFQ at home and then return to research staff on visit 3. Adult/parent participants recruited in Pohnpei and Palau are not asked to complete the FFQ since the FFQ was only validated for use among adults living in Guam and the Mariana Islands, thus it would not adequately capture dietary intake among adult living in other Pacific islands. Once the FFQs are collected, nutrient intakes are evaluated using the Dietary Reference Intakes, following the procedures recommended by the Institute of Medicine [54–59]. Adherence to United States Department of Agriculture (USDA) recommendations will be determined by calculating an 8-point USDA score described by Dixon and colleagues [60].

### 24-Hour diet recall

For adults and children recruited in Pohnpei and Palau, a 24-h dietary recall is conducted using the Modified 3-Pass Method [61] to determine dietary intake. Dietary intake of children is reported by parents. A project staff member, trained by a registered dietitian, asks the participants to list all foods eaten on the previous day, going from midnight to the following midnight; and foods are recorded on the 24-h diet recall form. When the participant is done recalling the foods eaten, the interviewer goes through all foods to determine when the food was consumed, how much of each food was consumed, and other specific information. Once the recall is completed, the foods are converted to grams consumed. A food consumption table based on the USDA Standard Release data, supplemented with Pacific food information, is applied to compute daily intakes of dietary components, including food groups, macronutrients, dietary fiber, and common nutrients. In addition to providing a snapshot of the foods consumed by both adults and children in Palau and Pohnpei, the data collected from the 24-h diet recalls will be used to generate food lists of commonly eaten foods and later used to develop specific food frequency questions for both Pohnpei and Palau.

### Food logs

Dietary intake of children in Guam is assessed by 2-day food logs reported by parents. Food logs are used to assess energy, nutrient, and food group intake of the child. The format and methods used for the food logs have been adapted from previous studies [37, 62]. Parents are asked to complete the food log for their children on two randomly assigned non-consecutive days, which includes weekdays and weekend days. Assignment of recording days is based on the day of the family’s first visit (Monday – Saturday). Standard techniques are used to improve accuracy of information recorded in the food logs [63]. Parents are instructed in record keeping techniques with the aid of food models, service ware, and utensils; and they are provided with a tool kit of calibrated utensils (i.e., measuring cups and spoons) which they take home and keep, as additional compensation. Research staff follow-up with reminder telephone calls. During visit 2 (for Palau and Pohnpei) or 3 (for Guam), staff review the food log with the parents for completeness of food entries, portion size estimation, food preparation methods, and accuracy of recording data. The food logs are entered by research staff onto the Pacific Tracker 3.1 (PacTrac3.1) and then sent to the University of Hawaii Cancer Center Shared Resources (UHCC) for quality control. Data from the PacTrac3 are used to estimate food groups, nutrients, and the healthy eating index using a food composition database developed by the UHCC for use in the Pacific region [62, 64].

### Accelerometers

Physical activity (PA) is measured with Actical accelerometers (Z series, Phillips Respironics Inc; Murrysville PA), which is a small, lightweight, water-resistant, omni-directional device designed to measure movement in multiple planes and provide data on intensity, frequency, and duration of activities in both children and adults [65]. The devise is worn on the participant’s non-dominant wrist attached with an Ident-A-Band (tear-resistant; Hollister; San Fernando CA) plastic band. A trained research staff member places an accelerometer on both the child and the parent, and participants are asked to wear the device daily (without removal) until it is removed by research staff 6 days later. Parents are instructed on proper use, how to replace the band if necessary, and assured that the device could be worn while sleeping, bathing, or swimming. Parents are also supplied with additional bands to be replaced at home if needed. Research staff show participants how to properly remove the Actical from wrist (by simply cutting the plastic band with scissors) and give adult participants a card to fill out (date and time of removal, date and time of reapplication of device if at all, and reason for removal) in the event they remove the Actical device. Once the Actical device is returned, research staff retrieve data using the manufacturer’s software (Actical version 3.0) with output activity in counts/minute.

### Acanthosis nigricans assessment

Acanthosis nigricans (AN), a skin indicator of hyperinsulinemia, an important risk factor for type 2 diabetes [66], is associated with obesity and is an indicator of clinical significance [39]. AN is assessed in both adults and children in two ways. First, trained research staff conduct a visual inspection of each participants’ neck; and second, a photo is taken of the neck. For the visual inspection of both adults and children, participants are asked to remove their hats, scarves, sweaters, and any hair accessories or necklaces to prevent obstruction of view to the neck. The research staff member wears sterile gloves, and then removes all surface dirt from the back of the neck area by using an alcohol swab. The research staff member then examines and observes the neck area, starting from the back before looking around to the sides and front of the participant, looking for a brown, velvety, sometimes veracious (wart-like) discoloration of the skin around the collar area. The severity of AN is recorded using the Acanthosis Nigricans Screening Scale [39]. After the participant gives verbal assent, the research staff member takes a photo of the back of the neck and side of the neck in front of a white background and a greyscale in the background as well. Participants’ faces are not photographed and are not identifiable; thus, insuring confidentiality. Photos are uploaded onto the PICCAH secured database and identified with the participant ID number. Photos are also given to a physician for assessment.

### Blood pressure

Resting blood pressure (BP) is measured by a trained research staff member with System 5 Multi Cuff sphygmomanometers (American Diagnostic Corporation, Hauppauge, NY, USA) and Littmann stethoscopes (3 M™, Saint Paul, MN, USA). Blood pressure is measured in a seated position two to three times with five minutes of rest between readings.

### Blood collection

On Guam, blood samples are collected by a certified phlebotomist, with participants having fasted for at least 12 h, and sent to a CLIA certified local laboratory (Diagnostic Laboratory Services). Fasting plasma glucose (FPG) is measured quantitatively by the enzymatic reference method with hexokinase while glycated hemoglobin (HbA1C) was assessed immunoturbidimetrically. For lipid profiles, total cholesterol, triglycerides, and HDL are assessed directly using standard enzymatic colorimetric method, while LDL and non-HDL are calculated values. One vial is sent to the Hawaii DLS location to assess fasting insulin via electrochemiluminescence immunoassay. All samples are analyzed using the Cobas® 6000 analyzer series (Roche Diagnostics, Basel, Switzerland). Aliquots of serum and plasma are stored in a Lexicon II Ultra-Low Temperature -80 °C freezer (ESCO, Hatboro, PA, USA) for participants who consent to long-term enrollment. On Pohnpei and Palau, blood samples are collected and processed at the regional community health center. Glucose is measured via finger stick method using a Precichek Autocode Blood Glucose Monitoring System AC-300. HbA1C is assessed using immunoassay methods with a DCA 2000 + Analyzer (Siemens/Bayer, Germany). Lipid profile is assessed using the Piccolo Xpress portable diagnostic analyzer (Abaxis, Inc., Union City, CA, USA). Due to laboratory constraints, insulin cannot be measured in Pohnpei or Palau.

### Data entry

The survey and measurement data collected from participants are entered into a custom secured local PICCAH database. The database data is analyzed for accuracy, consistency, and reporting overall project efforts.

## Discussion

The purpose of this paper is to present the rationale and design of the PICCAH Study being conducted in Guam, Pohnpei, and Palau. This study is innovative as it will expand knowledge of the extent of the interrelated cardiometabolic conditions such as obesity diabetes, and CVD and related risk factors in a subset of Pacific Islanders residing in the USAPI region, which is a vastly underrepresented and understudied group in population health research. The data collected will be among the first to look at health indicators across generations of USAPI populations and will inform future research projects.

In addition to collecting critical information on the cardiometabolic risk factors among children and adults in Guam, Pohnpei, and Palau, we are also learning valuable lessons on acceptable recruitment and data collection practices with these indigenous groups, and best practices to engage them in health research. As mentioned earlier, the initial intent was to use a stratified sampling framework to recruit a representative sample of children in Guam using the Guam EHDI Network; a strategy that relied on letters and phone calls to recruit participants. This strategy was shown to be ineffective for several reasons including: 1) the Guam EHDI Network did not have an up-to-date listing of the older children in their database; 2) the target population did not respond well to letters in the mail and perceived it as impersonal junk mail; and 3) the target population did not want to participate in the project unless they had a personal connection to someone else – either friend, family member or community leader – who knew about the project in some way. This finding is supported by Chung-Do and colleagues [67], who reported that when engaging Pacific Islanders in research, the most common element needed for success was the investment in long-term relationships and the human connection because “relationships are the most important”. Therefore, we switched to community-based, convenience sampling to recruit participants proved to be quite successful. As discussed earlier, some of the basic recruitment components that helped facilitate recruitment of the indigenous Pacific islander participants included: 1) creating relationships with various community based groups, institutions, organizations, businesses, etc. with similar goals or similar target audiences for a wide-ranging approach; 2) establishing presence and trust through community outreach and awareness, such as participating in community health fairs at local community centers, churches and shopping malls, where research staff members provided health information, promotional items for children, and free assessments of blood pressure; and 3) partnering with multiple community champions for advocacy and coordination.

Another goal of the PICCAH study is to help build the research capacities in this region. An important strategy to help build capacity in Pohnpei was the successful collaboration between the UOG researchers and the College of Micronesia/Federated States of Micronesia(COM/FSM), to establish a Federal Wide Assurance (FWA) for the Protection of Human Subjects and to establish an IRB at COM/FSM. The FWA was approved in December 2016. The next step was to assist in establishing the IRB. PICCAH provided support for COM/FSM officials to be trained through the Office for Human Research Protections (OHRP) Research Community Forum in preparation for developing the IRB at COM/FSM. In November 2016, the COM-FSM IRB was established by the President’s Cabinet, members were selected, and policies were drafted. The COM-FSM IRB Chairperson registered the IRB with the US DHHS and on 12/8/2017, and the College then received notification of acceptance by the OHRP and HHS. The achievement of the FWA and the establishment of the IRB are key steps in capacity building for the Federated States of Micronesia, as this was the first time it was done in the FSM.

Another positive outcome of the capacity building efforts in Pohnpei is the provision of a -80° freezer to COM/FSM to store biospecimens. The freezer is operational and the Pohnpei researcher has been able to utilize the freezer for specimen storage for students/researchers participating in a National Institute of Diabetes and Digestive and Kidney Diseases Short-Term Research Experience Program to Unlock Potential (NIDDK STEP-UP), held annually at the COM/FSM. Prior to the U24 PICCAH efforts, there was no capacity for quality biospecimen storage in Pohnpei.

Research capacity has been built through the incorporation of STEM student learning research opportunities. The University of Guam is a partner in the NIH U-54 Building Infrastructure Leading to Diversity Enhancing Cross Disciplinary Infrastructure and Training at Oregon (BUILD EXITO). PICCAH is a Research Learning Community and enables students in STEM majors to participate in research activities. Three of the PICCAH investigators are Research Mentors for the NIH BUILD EXITO program, and students have contributed to the work on this study. Recently one former BUILD research student went on to complete graduate education and worked in Cancer Health Disparity Research after graduating from UOG. Several other students who were active in this project are currently in STEM graduate education programs. In Pohnpei, students from STEM programs at the college have participated in the data collection activities. Provision of student research experiences in Guam and Micronesia will help increase capacity in our region.

Over time, both the quantitative and qualitative information collected in this study will be used to refine health messages and intervention strategies, as well as improve the quality of health care practices relevant to Pacific Islanders. Capacity building efforts may lead to increased research among our Pacific Islander populations in our region.

## Supplementary Information

**Additional file 1.**

**Additional file 2.**

## Data Availability

The datasets generated and/or analyzed during the current study are not publicly available as we are still in the process of collecting and entering data. However, data will be available from the corresponding author after completion of data entry and analysis on reasonable request.

## Abbreviations

AN: Acanthosis nigricans
BP: Blood pressure
CHC: Community Health Center
COM: College of Micronesia-FSM
CVD: Cardiovascular disease
DM: Diabetes mellitus
EHDI: Early Hearing Detection & Intervention
FWA: Federal Wide Assurance
FFQ: Food frequency questionnaire
FPG: Fasting plasma glucose
FSM: Federated States of Micronesia
HbA1C: Glycated hemoglobin
MOH: Palau Ministry of Health
NIMHD: National Institute of Minority Health Disparities
NCD: Non-communicable disease
OWOB: Overweight and obesity
PA: Physical activity
PacTrac3.1: Pacific Tracker 3.1 Diet Analysis Software
PICCAH: Pacific Islands Cohort on Cardiometabolic Health
PIHOA: Pacific Island Health Officers’ Association
USDA: United States Department of Agriculture
UHCC: University of Hawaii Cancer Center
SES: Socioeconomic status
UOG-CEDDERS: University of Guam Center for Excellence for Developmental Disabilities Education and Research Services
USAPI: United States Affiliated Pacific Island
