# Supplementary material for: Pacific Islands Cohort on Cardiometabolic Health Study: rationale and design
Source: BMC Public Health. 2022 Jul 27;22:1428. doi: 10.1186/s12889-022-13783-9 (PMC9326143; doi:10.1186/s12889-022-13783-9)
Supplement: Supplementary file 1 — Additional file 1. [file 12889_2022_13783_MOESM1_ESM.pdf]

Date: ______________ Interviewer Initials: __________ ID#: __________ FORM U24-22A

ACANTHOSIS NIGRICANS (ADULT)

Instructions: Rate and circle using a black/blue pen the severity of Acanthosis nigricans on the back of the neck using the screening scale below.

Neck Severity Rating:	0	1	2	3	4
Comments:					

Acanthosis Nigricans Screening Scale

(Burke JP, Hale DE, Hazuda HP, Stern MP. 1999. A quantitative scale of acanthosis nigricans. Diabetes Care 22:1655–1659.)

Neck Severity Rating	Neck Severity	Description
0	Absent	Not detectable on close inspection.
1	Present	Clearly present on close visual inspection, not visible to the casual observer, extent not measurable
2	Mild	Limited to the base of the skull, does not extend to the lateral margins of the neck (usually <3 inches in breadth).
3	Moderate	Extending to the lateral margins of the neck (posterior border of the sternocleidomastoid) (usually 3-6 inches), should not be visible when the participant is viewed from the front.
4	Severe	Extending anteriorly (>6 inches), visible when the participant is viewed from the front.

CHRS#: **21-120**
Approval Date: 10/22/2021
Expiration Date: 10/22/2022

Date: ______________ Interviewer Initials: __________ ID#: __________ FORM U24-22B

ACANTHOSIS NIGRICANS (CHILD)

Instructions: Rate and circle using a black/blue pen the severity of Acanthosis nigricans on the back of the neck using the screening scale below.

Neck Severity Rating: 0 1 2 3 4

Comments: ____

____

Acanthosis Nigricans Screening Scale

(Burke JP, Hale DE, Hazuda HP, Stern MP. 1999. A quantitative scale of acanthosis nigricans. Diabetes Care 22:1655–1659.)

Neck Severity Rating	Neck Severity	Description
0	Absent	Not detectable on close inspection.
1	Present	Clearly present on close visual inspection, not visible to the casual observer, extent not measurable
2	Mild	Limited to the base of the skull, does not extend to the lateral margins of the neck (usually <3 inches in breadth).
3	Moderate	Extending to the lateral margins of the neck (posterior border of the sternocleidomastoid) (usually 3-6 inches), should not be visible when the participant is viewed from the front.
4	Severe	Extending anteriorly (>6 inches), visible when the participant is viewed from the front.

CHRS#: **21-120**
Approval Date: 10/22/2021
Expiration Date: 10/22/2022

Date: ______________ Interviewer Initials: ________ ID#: __________ FORM U24-XX

INFORMATION ABOUT YOUR CHILD AND HOUSEHOLD

Please complete all 10 pages of this form. When completing this form, consider the child who will participate in the Pacific Islands Cohort on Cardiometabolic Health study. Thank you!

HOUSEHOLD COMPOSITION

1. What is the relationship to this child?

☐ Birth mother
☐ Birth father
☐ Step mother
☐ Step father
☐ Adoptive mother
☐ Adoptive father
☐ Legal guardian, caregiver, other: If related, please indicate the relationship (e.g., grandmother, uncle, sibling) _______________________

2. Who currently lives in the child's household and how are they related to your child? Mark all that apply and specify how many for each.

☐ Mother: ______	☐ Father: ______
☐ Sister: ______	☐ Brother: ______
☐ Grandmother: ______	☐ Grandfather: ______
☐ Aunt: ______	☐ Uncle: ______
☐ Cousin: ______	☐ Friend: ______

☐ Other (please specify): ____________________________

Date: ______________ Interviewer Initials: _______ ID#: __________ FORM U24-XX

3. Please tell us about **OTHER** children (for example: siblings, cousins, friends) who live with your child on a regular basis.

Please specify below whether the child is a boy or a girl and the age of the child.

	Boy	Girl	Age
Child 1	☐	☐	
Child 2	☐	☐	
Child 3	☐	☐	
Child 4	☐	☐	
Child 5	☐	☐	
Child 6	☐	☐	
Child 7	☐	☐	
Child 8	☐	☐	
Child 9	☐	☐	
Child 10	☐	☐	

CHILD INFORMATION

1. Your child's grade in school.

☐ Head start ☐ Daycare ☐ Preschool

☐ Kindergarten ☐ Elementary ☐ None

2. Do you consider your child to be of Cuban, Mexican, Puerto Rican, South or Central American, or other Spanish cultural heritage?

☐ Yes ☐ No

Date: ______________ Interviewer Initials: ________ ID#: __________ FORM U24-XX

3. Which category(s) below best describes your child? Mark all that apply.

☐ **Black or African American** – A person having origins of any of the original peoples of Africa.

☐ **White** – A person having origins in any of the original peoples of Europe, the Middle East, or North Africa.

☐ **American Indian or Alaskan Native** – A person having origin in any of the original peoples of North or South America (including Central America), and who maintains tribal affiliation or community attachment.

Please specify the one(s) you most identify with. Mark all that apply.

☐ Athabascan	☐ Siberian
☐ Cup'ik	☐ Yup'ik
☐ Inupiaq	☐ Other (please describe): __________________

☐ **Asian**

Please specify the one(s) you most identify with. Mark all that apply.

☐ Cambodian	☐ Japanese	☐ Pakistani
☐ Chinese	☐ Korean	☐ Thai
☐ Filipino	☐ Malaysian	☐ Vietnamese
☐ Indian	☐ Other (please describe): ______________________	

☐ **Native Hawaiian or other Pacific Islander**

Please specify the one(s) you most identify with. Mark all that apply.

☐ Chamorro	☐ Kosraean	☐ Pohnpeian	☐ Tokelaun
☐ Carolinian	☐ Marshallese	☐ Samoan	☐ Tahitian
☐ Chuukese	☐ Native Hawaiian	☐ Tongan	☐ Yapese
☐ Kiribati	☐ Palauan	☐ Other (please describe):____________	

4. Which cultural group does your child MOST identify with? ____________________________

5. What language(s) does your child speak? ____

6. What language does your child most often speak at home? ___________________________

Date: _____________ Interviewer Initials: _______ ID#: _________ FORM U24-XX

EARLY LIFE OF YOUR CHILD

1. Child's Birth Weight: ______ **lbs.** and _____ **oz.** ☐ Unknown

2. Child's Birth Length: _________ **in.** ☐ Unknown

3. How was your child born?

   ☐ Vaginally ☐ Cesarean Section ☐ Unknown

4. Did your child have any complications at birth?

   ☐ Yes (please describe):____________________ ☐ No ☐ Unknown

5. Was your child ever breastfed or fed breastmilk?

   ☐ Yes ☐ No **(skip to question 6)**
   ☐ Unknown ☐ Other (please describe): ____________________

   If yes, how old was your child when he/she completely stopped breastfeeding or being fed breastmilk?

   ☐ ________ Months of age ☐ Still Breastfeeding ☐ Unknown

6. Was your child ever fed formula?

   ☐ Yes ☐ No **(skip to question 7)**
   ☐ Unknown ☐ Other (please describe): _____________________

   If yes, how old was the child when he/she was first fed formula?

   ☐ _________ Months of age ☐ Since Birth ☐ Unknown

Date: _____________ Interviewer Initials: _______ ID#: _________ FORM U24-XX

If your child was fed formula, how old was your child when he/she completely stopped drinking formula?

☐ ________ Months of age ☐ Still formula fed ☐ Unknown

7. How old was the child when he/she was first fed anything other than breastmilk or formula? (This includes juice, cow's milk, sugar water, baby food, or anything else that the child might have been given, even water)

☐ ______ Months of age ☐ Unknown

MEDICAL INFORMATION

1. What type of health insurance do you currently have? Mark only one.

☐ Insurance through a current or former employer (please specify): ____________________
☐ Medicare
☐ Medicaid, medical assistance, MIP or any kind of government assistance plan for low incomes or disability
☐ Tricare or other military health care
☐ Other health care coverage (please describe): ____________________
☐ I do not have health insurance
☐ Unknown

2. Is your child under the same health care coverage?

☐ Yes ☐ No (please describe): _________________ ☐ Unknown

3. Does your child have any current medical/behavioral conditions diagnosed by a doctor?

☐ Yes ☐ No ☐ Unknown

If yes, please specify: ____

____

4. Has a doctor or nurse ever told you that your child has asthma?

☐ Yes ☐ No ☐ Unknown

Date: _____________ Interviewer Initials: _______ ID#: _________ FORM U24-XX

PARENT INFORMATION

1. What is your current marital status. Mark only one.

☐ Married ☐ Widowed

☐ Divorced ☐ Single and NOT living with boyfriend, girlfriend, or partner

☐ Separated ☐ Single and living with boyfriend, girlfriend, or partner

☐ Other (please describe): ______________________________

2. What is the highest grade or year of school you completed?

☐ Never attended school or only attended kindergarten

☐ Grade 12 or GED (High school graduate)

☐ Grades 1 up to 8 (Elementary to middle school)

☐ College or technical school 1-3 years

☐ Grades 9 up to 11 (Some high school)

☐ College 4 or more years (college graduate)

3. What state-hamlet do you reside in Palau?

State: ______________________________ Duration: _________ years

4. Have you resided in other hamlets in Palau?

☐ Yes (Please specify:) ☐ No

Hamlet: Duration:

_________________________ _________ years

_________________________ _________ years

_________________________ _________ years

Date: ______________ Interviewer Initials: ________ ID#: __________ FORM U24-XX

5. Have you ever been affiliated with the U.S. military?

☐ Yes ☐ No **(skip to question 7)** ☐ Unknown

If yes, what is your affiliation with the U.S. military?

☐ I currently serve in the U.S. armed forces.

☐ I am a veteran.

☐ I (am/was) a military dependent.

☐ I (work/have worked) as a civilian on military grounds.

☐ Other (please specify):__________________________

6. Years affiliated with the U.S. military.

☐ _____ Total years with U.S. military ☐ _____ Years with U.S. military in Palau

Date: ______________ Interviewer Initials: ________ ID#: __________ FORM U24-XX

7. Please select which cultural group you MOST identify with. Mark only one. *(If you identify with a group that is made up of subgroups, please mark the one subgroup that you most identify with.)*

☐ **Black or African American** – A person having origins of any of the original peoples of Africa.

☐ **White** – A person having origins in any of the original peoples of Europe, the Middle East, or North Africa.

☐ **American Indian or Alaskan Native** – A person having origin in any of the original peoples of North or South America (including Central America), and who maintains tribal affiliation or community attachment.

Please specify the one you most identify with. Mark all that apply.

☐ Athabascan	☐ Siberian
☐ Cup'ik	☐ Yup'ik
☐ Inupiaq	☐ Other (please describe): __________________

☐ **Asian**

Please specify the one you most identify with. Mark all that apply.

☐ Cambodian	☐ Japanese	☐ Pakistani
☐ Chinese	☐ Korean	☐ Thai
☐ Filipino	☐ Malaysian	☐ Vietnamese
☐ Indian	☐ Other (please describe): _____________________	

☐ **Native Hawaiian or other Pacific Islander**

Please specify the one you most identify with. Mark all that apply.

☐ Chamorro	☐ Kosraean	☐ Pohnpeian	☐ Tokelaun
☐ Carolinian	☐ Marshallese	☐ Samoan	☐ Tahitian
☐ Chuukese	☐ Native Hawaiian	☐ Tongan	☐ Yapese
☐ Kiribati	☐ Palauan	☐ Other (please describe):____________	

Date: ____________ Interviewer Initials: _______ ID#: _________ FORM U24-XX

8. Your current employment status? Mark all that apply.
   - ☐ Employed for wages/salary (full-time/part-time/seasonal)
   - ☐ Out of work for more than 1 year
   - ☐ Out of work for less than 1 year
   - ☐ Retired
   - ☐ Self-employed
   - ☐ A Homemaker
   - ☐ A student
   - ☐ Unable to work

9. Do you currently have more than one job at this time?
   - ☐ Yes
   - ☐ No

10. Based on everyone that lives under one roof or house, what is the annual household income from all sources over the past 12 months?
    - ☐ Under $10,000
    - ☐ From $10,000 to less than $20,000
    - ☐ From $20,000 to less than $35,000
    - ☐ From $35,000 to less than $60,000
    - ☐ From $60,000 to less than $75,000
    - ☐ $75,000 or more
    - ☐ No Response

11. What language(s) do you speak? ____

12. What language do you speak most often at home? _________________________________

13. In what city or country were you born? ____

14. How many years have you lived here in Palau? _______________

FOOD SECURITY/AVAILABILITY

1. In the past 12 months, how often did your money for food run out before the end of the month?
   - ☐ Never
   - ☐ Seldom
   - ☐ Sometimes
   - ☐ Most times
   - ☐ Always
   - ☐ Unknown
   - ☐ No Response

Date: ____________ Interviewer Initials: _______ ID#: _________ FORM U24-XX

2. In the past 12 months, how often did your money for household utilities (e.g. water, fuel oil, electricity) run out before the end of the month? Mark which applies to you.
   - ☐ Never
   - ☐ Seldom
   - ☐ Sometimes
   - ☐ Most times
   - ☐ Always
   - ☐ Unknown
   - ☐ No Response

3. In the past 12 months, did you receive assistance to pay for food (e.g. food stamps, WIC coupons)?
   - ☐ Yes
   - ☐ No
   - ☐ No Response

   If yes, which benefits does this household receive?
   - ☐ EBT/SNAP/NAP (formerly Food Stamps)
   - ☐ Food Assistance (Food Bank/Food Pantries or Commodity foods)
   - ☐ WIC benefits
   - ☐ Free or reduced-cost breakfasts or lunches at schools
   - ☐ Don't known
   - ☐ Not applicable

RELIGION

1. What is your religious affiliation?
   - ☐ Baptist
   - ☐ Buddhist
   - ☐ Catholic
   - ☐ Episcopalian
   - ☐ Evangelical Covenant
   - ☐ Mormon/ Latter-day Saints
   - ☐ Moravian
   - ☐ Muslim
   - ☐ Pentecostal
   - ☐ Protestant
   - ☐ Russian Orthodox
   - ☐ Other (please describe): _________________
   - ☐ None
   - ☐ No Response

2. How often do you engage in religious activities or events with your religious community?
   - ☐ ___ per week
   - ☐ ___ per month
   - ☐ Do not attend
   - ☐ No Response

Date: ____________ Interviewer Initials: ___________ ID#: _______________

MEDICAL CONDITIONS/ HISTORY (ADULTS)

Has a medical doctor ever told you that you had/have…	If yes, how old were you when you were first told?	Do/Did either of your parents have…
**1. Diabetes?**<br>☐ Yes ☐ No<br><br>If yes, what type?<br>□ Type I<br>□ Type II<br>□ Gestational	_________ years	Mom: ☐ Yes ☐ No<br>Dad: ☐ Yes ☐ No
**2. Heart Attack?**<br>☐ Yes ☐ No	_________ years	Mom: ☐ Yes ☐ No<br>Dad: ☐ Yes ☐ No
**3. Stroke?**<br>☐ Yes ☐ No	_________ years	Mom: ☐ Yes ☐ No<br>Dad: ☐ Yes ☐ No
**4. Hypertension?**<br>☐ Yes ☐ No	_________ years	Mom: ☐ Yes ☐ No<br>Dad: ☐ Yes ☐ No

CHRS#: 21-120 | Approval Date: 10/22/2021 | Expiration Date: 10/22/2022

Date: ____________ Interviewer Initials: ____________ ID#: ______________

Have you ever taken any of the following medications at least two times per week (for one month or longer)?		If yes, how many years have you ever taken them?	
**Aspirin** (Anacin, Bufferin, Bayer, Excedrin, Ecotrin or other)	☐ No<br>☐ Yes, but not now<br>☐ Yes, currently	☐ 1 year or less<br>☐ 2 to 3 years<br>☐ 4 to 5 years	☐ 6 to 10 years<br>☐ 11 to 15 years<br>☐ 16 years or more
**Acetaminophen** (Tylenol, Aspirin-Free Anacin or other)	☐ No<br>☐ Yes, but not now<br>☐ Yes, currently	☐ 1 year or less<br>☐ 2 to 3 years<br>☐ 4 to 5 years	☐ 6 to 10 years<br>☐ 11 to 15 years<br>☐ 16 years or more
**Celebrex** (Celecoxib), **Vox** (forecoxa), **Extra** (Valdecoxib)	☐ No<br>☐ Yes, but not now<br>☐ Yes, currently	☐ 1 year or less<br>☐ 2 to 3 years<br>☐ 4 to 5 years	☐ 6 to 10 years<br>☐ 11 to 15 years<br>☐ 16 years or more
**Other Pain Relief Medication** (Motrin Ibuprofen, Advil, Aleve, Naprosyn, Indocin,	☐ No<br>☐ Yes, but not now<br>☐ Yes, currently	☐ 1 year or less<br>☐ 2 to 3 years<br>☐ 4 to 5 years	☐ 6 to 10 years<br>☐ 11 to 15 years<br>☐ 16 years or more
**Water Pills for High Blood Pressure or Other Reasons** (Hydrochlorothiazide, Maidie, Furosemide, Lasix or other)	☐ No<br>☐ Yes, but not now<br>☐ Yes, currently	☐ 1 year or less<br>☐ 2 to 3 years<br>☐ 4 to 5 years	☐ 6 to 10 years<br>☐ 11 to 15 years<br>☐ 16 years or more
**High Blood Pressure Medication** (Verapamil, Norvasc, Prineville, Cozier, Procardia, Atenolol, Metoprolol, Lutenin, Vaster other other)	☐ No<br>☐ Yes, but not now<br>☐ Yes, currently	☐ 1 year or less<br>☐ 2 to 3 years<br>☐ 4 to 5 years	☐ 6 to 10 years<br>☐ 11 to 15 years<br>☐ 16 years or more
**High Cholesterol Medication** (Lipitor, Evictor, Zocor, Prava Chol, Loped or other)	☐ No<br>☐ Yes, but not now<br>☐ Yes, currently	☐ 1 year or less<br>☐ 2 to 3 years<br>☐ 4 to 5 years	☐ 6 to 10 years<br>☐ 11 to 15 years<br>☐ 16 years or more
**Peptic Ulcer Medication** (Tagamet, Zantac, Pepcid, Prefaced, Prilosec or other)	☐ No<br>☐ Yes, but not now<br>☐ Yes, currently	☐ 1 year or less<br>☐ 2 to 3 years<br>☐ 4 to 5 years	☐ 6 to 10 years<br>☐ 11 to 15 years<br>☐ 16 years or more
**Pill for Diabetes** (Glucophage, Glyburide, Glucotrol, Avandia, Actos or other)	☐ No<br>☐ Yes, but not now<br>☐ Yes, currently	☐ 1 year or less<br>☐ 2 to 3 years<br>☐ 4 to 5 years	☐ 6 to 10 years<br>☐ 11 to 15 years<br>☐ 16 years or more
**Insulin Shots for Diabetes**	☐ No<br>☐ Yes, but not now<br>☐ Yes, currently	☐ 1 year or less<br>☐ 2 to 3 years<br>☐ 4 to 5 years	☐ 6 to 10 years<br>☐ 11 to 15 years<br>☐ 16 years or more
**Allergy Pill or Shots** (Antihistamines such as Claritin, Allegra, Zyrtec, Benadryl or other)	☐ No<br>☐ Yes, but not now<br>☐ Yes, currently	☐ 1 year or less<br>☐ 2 to 3 years<br>☐ 4 to 5 years	☐ 6 to 10 years<br>☐ 11 to 15 years<br>☐ 16 years or more
**Asthma Medication Pills or Inhalers** (Albuterol, Nasacort, Proventil, Theophylline or other)	☐ No<br>☐ Yes, but not now<br>☐ Yes, currently	☐ 1 year or less<br>☐ 2 to 3 years<br>☐ 4 to 5 years	☐ 6 to 10 years<br>☐ 11 to 15 years<br>☐ 16 years or more
**Evita** (Raloxifene)	☐ No<br>☐ Yes, but not now<br>☐ Yes, currently	☐ 1 year or less<br>☐ 2 to 3 years<br>☐ 4 to 5 years	☐ 6 to 10 years<br>☐ 11 to 15 years<br>☐ 16 years or more
**Actonel** (Iduronate), **Fosamax** (Alendronate) for brittle bones	☐ No<br>☐ Yes, but not now<br>☐ Yes, currently	☐ 1 year or less<br>☐ 2 to 3 years<br>☐ 4 to 5 years	☐ 6 to 10 years<br>☐ 11 to 15 years<br>☐ 16 years or more
**Steroids** (prednisone, hydrocortisone, or other)	☐ No<br>☐ Yes, but not now<br>☐ Yes, currently	If yes, when was your last dose taken?	

List any other medications you take regularly:

CHRS#: 21-120 | Approval Date: 10/22/2021 | Expiration Date: 10/22/2022

Date: ____________ Interviewer Initials: ___________ ID#: _______________

CULTURE (ADULTS)

Below are questions about your attitude and beliefs on **your group's** culture and lifestyle. Please read each question carefully and circle the response that best describes you.

Your Group's Heritage and Lifestyle					
1) How <u>knowledgeable</u> are you of **your group's** traditional culture and lifestyle?	Not at all knowledgeable	Somewhat not knowledgeable	Neutral or no response	Somewhat knowledgeable	Very Knowledgeable
2) How <u>involved</u> are you in **your group's** traditional culture and lifestyle?	Not at all involved	Somewhat not involved	Neutral or no response	Somewhat involved	Very involved
3) How do you <u>feel toward</u> **your group's** traditional culture and lifestyle?	Very Negative	Somewhat negative	Neutral or no response	Somewhat positive	Very Positive
4) How often do you <u>associate</u> with people of **your group's** traditional culture and lifestyle?	Not at all	Very little of the time	Neutral or no response	Somewhat often	Most of the time

Below are questions about your attitude and beliefs on **U.S. Mainland** culture and lifestyle. Please read each question carefully and circle the response that best describes you.

U.S. Mainland Heritage and Lifestyle					
1) How <u>knowledgeable</u> are you of **U.S. Mainland** culture and lifestyle?	Not at all knowledgeable	Somewhat not knowledgeable	Neutral or no response	Somewhat knowledgeable	Very Knowledgeable
2) How <u>involved</u> are you in **U.S. Mainland** culture and lifestyle?	Not at all involved	Somewhat not involved	Neutral or no response	Somewhat involved	Very involved
3) How do you <u>feel toward</u> the **U.S. Mainland** culture and lifestyle?	Very Negative	Somewhat negative	Neutral or no response	Somewhat positive	Very Positive
4) How often do you <u>associate</u> with people of **U.S. Mainland** culture and lifestyle?	Not at all	Very little of the time	Neutral or no response	Somewhat often	Most of the time

CHRS#: 21-120 | Approval Date: 10/22/2021 | Expiration Date: 10/22/2022

Date: ____________ Interviewer Initials: ___________ ID#: ______________

BETEL NUT USE (ADULTS)

1. How long has it been since you last visited a dentist or a dental clinic for any reason? Include visits to dental specialists, such as orthodontists.

☐ Within the past year (anytime less than 12 months ago)
☐ Within the past 2 years (1 year but less than 2 years ago)
☐ Within the past 5 years (2 years but less than 5 years ago)
☐ 5 or more years ago
☐ Don't know / Not sure
☐ Never
☐ Refused

2. How often do you brush your teeth?

☐ More than once per day ☐ Once per day ☐ Once per week
☐ Once per year ☐ Never ☐ Don't know/Not sure
☐ Refused

3. Do you chew betel nut?

☐ No **[go to Page 16]** ☐ Yes ☐ Don't know/Not sure ☐ Refused

4. How many times per day, week or month do you chew betel nut?

☐_____times daily ☐ _____times weekly ☐______ times monthly

☐ Don't know/Not sure ☐ Refused

5. Do you include lime when chewing betel nut?

☐ No ☐ Yes ☐ Don't know/Not sure ☐ Refused

CHRS#: 21-120 | Approval Date: 10/22/2021 | Expiration Date: 10/22/2022

Date: ____________ Interviewer Initials: ___________ ID#: _______________

BETEL NUT USE (ADULTS)

6. Do you include tobacco when chewing betel nut?

☐ No ☐ Yes (Identify type: ___________________)
☐ Don't know/Not sure ☐ Refused

7. Do you include pepper leaf when chewing betel nut?

☐ No ☐ Yes ☐ Don't know/Not sure ☐ Refused

8. Do you add alcohol to any of the components of your chew (nut, leaf, lime or tobacco)?

☐ No ☐ Yes ☐ Don't know/Not sure ☐ Refused

9. How long have you been chewing betel nut? ______ years

☐ Don't know/Not sure ☐ Refused

CHRS#: 21-120 | Approval Date: 10/22/2021 | Expiration Date: 10/22/2022

Date: ____________ Interviewer Initials: ___________ ID#: _______________

TOBACCO USE (ADULTS)

1. Have you smoked at least 100 cigarettes in your entire life?

   NOTE: “For cigarettes, do not include: electronic cigarettes (e-cigarettes, NJOY, Bluetip), herbal cigarettes, cigars, cigarillos, little cigars, pipes, bidis, kreteks, water pipes (hookahs) or marijuana.”

   ☐ Yes ☐ No ☐ Don’t know/ Not sure ☐ Refused

2. Do you now smoke cigarettes every day, some days, or not at all?

   ☐ Every day ☐ Some days ☐ Not at all
   ☐ Don't Know/ Not sure ☐ Refused

3. Do you currently use chewing tobacco, snuff, or snus every day, some days, or not at all?

   ☐ Every day ☐ Some days ☐ Not at all
   ☐ Don't Know/ Not sure ☐ Refused

CHRS#: 21-120 | Approval Date: 10/22/2021 | Expiration Date: 10/22/2022

Date: ___________ Interviewer Initials: __________ ID#: ______________

ALCOHOL USE (ADULTS)

Mark and specify (if needed) the answer that best describes your alcohol consumption.

1. During the past 30 days, how many days per week or per month did you have at least one drink of any alcoholic beverage such as beer, wine, a malt beverage or liquor?

   ☐_____days per week ☐_____ days in past 30 days

   ☐ No drinks in past 30 days **[go to next page]** ☐ Don't know/ Not sure

   ☐Refused

2. One drink is equivalent to a 12-ounce beer, a 5-ounce glass of wine, or a drink with one shot of liquor. During the past 30 days, on the days when you drank, about how many drinks did you drink on the average?

   (NOTE: A 40-ounce beer would count as 3 drinks, or a cocktail drink with 2 shots would count as 2 drinks.)

   ☐_____ Number of drinks ☐ Don't know/ Not sure ☐ Refused

3. Considering all types of alcoholic beverages, how many times during the past 30 days did you have X [X = 5 for men, X = 4 for women] or more drinks on an occasion?

   ☐ _____ Number of times ☐ None ☐ Don't know/ Not sure

   ☐ Refused

4. During the past 30 days, what is the largest number of drinks you had on any occasion?

   ☐_____ Number of drinks ☐ Don't know/ Not sure ☐ Refused

CHRS#: 21-120 | Approval Date: 10/22/2021 | Expiration Date: 10/22/2022

Date: ____________ Interviewer Initials: ___________ ID#: _______________

SLEEP (ADULTS)

1. What time do you usually go to bed? ___________ AM / PM

2. How long (in minutes) does it take you to fall asleep each night? ___________

3. What time do you usually get up in the morning? ___________ AM / PM

4. How many hours of actual sleep do you get at night? (This may be different than the number of hours you spend in bed) ___________

5. Do you have to take medicine (prescribed or over the counter) to help you sleep?

   ☐ No ☐ Yes

6. How would you rate your sleep quality overall?

   ☐ Very good ☐ Fairly good ☐ Fairly bad ☐ Very bad

CHRS#: 21-120 | Approval Date: 10/22/2021 | Expiration Date: 10/22/2022

Date: ____________ Interviewer Initials: ___________ ID#: ______________

OCCUPATIONAL EXPOSURE (ADULTS)

1. On the average, during the last year, how many hours in a day did you spend in the following sitting activities?

MARK ONLY ONE

	Never	Less than 1 hr.	1 to 2 hrs.	3 to 4 hrs.	5 to 6 hrs.	7 to 10 hrs.	11 hrs. or more
**Sitting in car, bus, truck or train**	☐	☐	☐	☐	☐	☐	☐
**Sitting at work**	☐	☐	☐	☐	☐	☐	☐
**Watching TV**	☐	☐	☐	☐	☐	☐	☐
**Sitting at meals**	☐	☐	☐	☐	☐	☐	☐
**Other sitting activities** (such as reading, playing cards, sewing, using a home computer)	☐	☐	☐	☐	☐	☐	☐

2. On the average, during the last year, how many hours in a week did you spend in the following activities?

MARK ONLY ONE

	Never	½ to 1 hr.	2 to 3 hrs.	4 to 6 hrs.	7 to 10 hrs.	11 to 20 hrs.	21 to 30 hrs.	31 hrs. or more
**Strenuous Recreational Activities** (such as running, jogging, bicycling on hills, soccer, tennis, swimming laps, aerobics, weightlifting)	☐	☐	☐	☐	☐	☐	☐	☐
**Strenuous Work** (such as moving heavy furniture, loading or unloading trucks, construction work, shoveling or equivalent labor)	☐	☐	☐	☐	☐	☐	☐	☐
**Moderate Recreational Activities** (such as brisk walking, golfing, bicycling on level ground, gardening, dancing, softball)	☐	☐	☐	☐	☐	☐	☐	☐
**Moderate Work** (such as housework, yard work, restaurant work, sales work, or equivalent moderate physical activity)	☐	☐	☐	☐	☐	☐	☐	☐

3. On the average, during the last year, how many times a week did you take part in vigorous physical activity (strenuous sports or work) long enough to work up a sweat?

☐ Never ☐ 1 time ☐ 2 times ☐ 3 times ☐ 4 times
☐ 5 times ☐ 6 times ☐ 7 times or more

CHRS#: 21-120 | Approval Date: 10/22/2021 | Expiration Date: 10/22/2022

Date: ____________ Interviewer Initials: ___________ ID#: _______________

DEPRESSION, ANXIETY & STRESS (ADULTS)

		Never	Sometimes	Often	Almost Always
1.	I found it hard to wind down.	☐	☐	☐	☐
2.	I was aware of dryness of my mouth.	☐	☐	☐	☐
3.	I could not seem to experience any positive feeling at all.	☐	☐	☐	☐
4.	I experienced breathing difficulty (e.g. Excessively rapid breathing, breathlessness in the absence of physical exertion.)	☐	☐	☐	☐
5.	I found it difficult to work up the initiative to do things.	☐	☐	☐	☐
6.	I tended to over-react to situations.	☐	☐	☐	☐
7.	I experienced trembling (e.g. In the hands)	☐	☐	☐	☐
8.	I felt that I was using a lot of nervous energy.	☐	☐	☐	☐
9.	I was worried about situations in which I might panic and make a fool of myself.	☐	☐	☐	☐
10.	I felt that I had nothing to look forward to.	☐	☐	☐	☐
11.	I found myself getting agitated.	☐	☐	☐	☐

CHRS#: 21-120 | Approval Date: 10/22/2021 | Expiration Date: 10/22/2022

Date: ____________ Interviewer Initials: ___________ ID#: _______________

		Never	Sometimes	Often	Almost Always
12.	I found it difficult to relax.	☐	☐	☐	☐
13.	I felt down-hearted and blue.	☐	☐	☐	☐
14.	I was intolerant of anything that kept me from getting on with what I was doing.	☐	☐	☐	☐
15.	I felt I was close to panic.	☐	☐	☐	☐
16.	I was unable to become enthusiastic about anything.	☐	☐	☐	☐
17.	I felt I wasn’t worth much as a person.	☐	☐	☐	☐
18.	I felt that I was rather touchy.	☐	☐	☐	☐
19.	I was aware of the action of my heart in the absence of physical exertion (e.g. Sense of heart rate increase, heart missing a beat)	☐	☐	☐	☐
20.	I felt scared without any good reason.	☐	☐	☐	☐
21.	I felt that life was meaningless.	☐	☐	☐	☐

CHRS#: 21-120 | Approval Date: 10/22/2021 | Expiration Date: 10/22/2022

Date: ____________ Interviewer Initials: ___________ ID#: ______________

PARENT-CHILD INTERACTION (ADULTS)

Definition: Physical activity includes sports and exercising as well as recreational activities such as walking, riding a bike, and gardening.

	Strongly Disagree	Disagree	Agree	Strongly Agree
1. I enroll my child in sports teams and clubs such as soccer, basketball, and dance.	1	2	3	4
2. I limit how long my child plays video games	1	2	3	4
3. I exercise or am physically active on a regular basis	1	2	3	4
4. I enjoy exercise and physical activity	1	2	3	4
5. I take my child to places where he/she can be active.	1	2	3	4
6. I limit how long my child can watch TV or DVDs each day (including educational and non-educational programs).	1	2	3	4
7. I encourage my child to use resources in our neighborhood to be active (such as the park and the school).	1	2	3	4
8. I enroll my child in community-based programs (such as Girls and Boys Club, YMCA) where he/she can be active.	1	2	3	4
9. I watch my child play sports or participate in other activities such as martial arts or dance.	1	2	3	4
10. I limit how long my child can use the computer for things other than homework (such as playing computer games and surfing the internet).	1	2	3	4
11. I encourage my child to be physically active by leading by example (by role modeling).	1	2	3	4
12. I find ways for my child to be active when school is out by, for example, enrolling him/her in summer camp and after school programs.	1	2	3	4

CHRS#: 21-120 | Approval Date: 10/22/2021 | Expiration Date: 10/22/2022

Date: ____________ Interviewer Initials: ___________ ID#: _______________

NUTRITION & PHYSICAL ACTIVITY (ADULTS)

Instructions: For each question, select the answer category that best fits your child or your family. It is important to indicate the most common or typical pattern for your family, and not what you would like to happen.

Family Meals	Never / Almost Never	Sometimes	Often	Very Often / Always
1. How often does your child eat breakfast, either at home or at school?	1	2	3	4
2. How often does your child eat at least one meal a day with at least one other family member?	1	2	3	4
**Family Eating Practices**	**Never / Almost Never**	**Sometimes**	**Often**	**Very Often / Always**
3. How often does your child eat while watching TV? [Includes meals or snacks]	1	2	3	4
4. How often does your family eat “fast food?”	1	2	3	4
**Food Choices**	**Never / Almost Never**	**Sometimes**	**Often**	**Very Often / Always**
5. How often does your family use packaged “ready-to-eat” foods? [Includes purchased frozen or on-the-shelf entrees, often designed to be microwaved]	1	2	3	4
6. How often does your child eat fruits and vegetables at meals or snacks? [Not including juice]	1	2	3	4
**Beverage Choices**	**Never / Almost Never**	**Sometimes**	**Often**	**Very Often / Always**
7. How often does your child drink soda pop or sweetened beverages? [Includes regular or diet soda pop, Kool--Aid, Sunny-D, Capri Sun, fruit or vegetable juice, caffeinated energy drinks (Monster/Red Bull), Powerade/Gatorade, etc.]	1	2	3	4
8. How often does your child drink low--fat milk for meals or snacks? [Includes 1% or skim dairy, flavored, soy, almond, etc.]	1	2	3	4

CHRS#: 21-120 | Approval Date: 10/22/2021 | Expiration Date: 10/22/2022

Date: ____________ Interviewer Initials: ___________ ID#: ______________

Restriction / Reward	Never / Almost Never	Sometimes	Often	Very Often / Always
9. How often does your family monitor the amount of candy, chips, and cookies your child eats?	1	2	3	4
10. How often does your family use candy, ice cream or other foods as a reward for good behavior?	1	2	3	4
**Screen Time**	**Never / Almost Never**	**Sometimes**	**Often**	**Very Often / Always**
11. How often does your child have less than 2 hours of “screen time” in a day? [Includes TV, computer, game system, or any mobile device with visual screens]	1	2	3	4
12. How often does your family monitor the amount of “screen time” your child has?	1	2	3	4
**Healthy Environment**	**Never / Almost Never**	**Sometimes**	**Often**	**Very Often / Always**
13. How often does your child engage in screen time in his/her bedroom?	1	2	3	4
14. How often does your family provide opportunities for physical activity?	1	2	3	4
**Family Activity**	**Never / Almost Never**	**Sometimes**	**Often**	**Very Often / Always**
15. How often does your family encourage your child to be physically active?	1	2	3	4
16. How often does your child do physical activities with at least one other family member?	1	2	3	4
**Child Activity**	**Never / Almost Never**	**Sometimes**	**Often**	**Very Often / Always**
17. How often does your child do something physically active when he/she has free time?	1	2	3	4
18. How often does your child participate in organized sports or physical activities with a coach or leader?	1	2	3	4
**Family Schedule / Sleep Routine**	**Never / Almost Never**	**Sometimes**	**Often**	**Very Often / Always**
19. How often does your child follow a regular routine for your child’s bedtime?	1	2	3	4
20. How often does your child get enough sleep at night?	1	2	3	4

CHRS#: 21-120 | Approval Date: 10/22/2021 | Expiration Date: 10/22/2022

Date: ____________ Interviewer Initials: ___________ ID#: _______________

PRENATAL AND PREGNANCY (ADULT)

***FEMALE PARTICIPANTS ONLY**

MALE PARTICIPANTS SKIP PAGES 25-26

1. Are you currently pregnant?

   ☐ No ☐ Yes ☐ Unknown

2. How many pregnancies have you had? _______ ☐ None **[go to page 29]**

3. How many live babies have you delivered? _________

4. Did you receive prenatal care during your pregnancy?

   ☐ No **[go to question 5]** ☐ Yes ☐ Don't Know/Not Sure

   If yes, where did you receive the care?

   ☐ Public Health ☐ Private Clinic ☐ Lay Midwife ☐ Don't Know/Not Sure

   If you received prenatal care, when did you begin prenatal care?

   ☐ In the first 3 months of pregnancy ☐ In the middle 3 months of pregnancy

   ☐ In the last 3 months of pregnancy ☐ Don't Know/Not Sure

5. How much weight did you gain during your pregnancy?

   ☐________ pounds ☐ Don't Know/Not Sure

CHRS#: 21-120 | Approval Date: 10/22/2021 | Expiration Date: 10/22/2022

Date: ____________ Interviewer Initials: ___________ ID#: _______________

PRENATAL AND PREGNANCY

6. How many weeks gestation were you when you delivered your baby?

☐ Prior to 32 weeks gestation

☐ 32-36 weeks gestation

☐ 37-40 weeks gestation

☐ After 40 weeks gestation

☐ Don't Know/Not Sure

7. Did you have any complications during your pregnancy?

☐ No **[go to question 8]** ☐ Yes ☐ Don't Know/Not Sure

If yes, indicate complication(s). Mark all that apply.

☐ Anemia ☐ High Blood Pressure ☐ Diabetes

☐ Heart Disease ☐ Preterm Labor ☐ Problems with the Placenta

☐ Premature rupture of membranes ☐ Psychological Disorders

☐ Other: ____________________________ ☐ Don't Know/Not Sure

8. What medications did you take during your pregnancy? Mark all that apply.

☐ No medication

☐ Prenatal vitamins

☐ Iron

☐ Other (Please specify):______________________________

☐ Don't Know/Not Sure

CHRS#: 21-120 | Approval Date: 10/22/2021 | Expiration Date: 10/22/2022

Date: ____________ Interviewer Initials: ___________ ID#: ______________

CULTURE (CHILD)

Below are questions about **your child's** attitude and beliefs on **his/her group's** culture and lifestyle. Please read each question carefully and circle the response that best describes your child.

Your Group's Heritage and Lifestyle

1) How knowledgeable is **your child** of **his/her group's** traditional culture and lifestyle?	Not at all knowledgeable	Somewhat not knowledgeable	Neutral or no response	Somewhat knowledgeable	Very Knowledgeable
2) How involved is **your child** in **his/her group's** traditional culture and lifestyle?	Not at all involved	Somewhat not involved	Neutral or no response	Somewhat involved	Very involved
3) How does **your child** feel toward **his/her group's** traditional culture and lifestyle?	Very Negative	Somewhat negative	Neutral or no response	Somewhat positive	Very Positive
4) How often does **your child** associate with people of **his/her group's** traditional culture and lifestyle?	Not at all	Very little of the time	Neutral or no response	Somewhat often	Most of the time

Below are questions about **your child's** attitude and beliefs on **U.S. Mainland** culture and lifestyle. Please read each question carefully and circle the response that best describes your child.

U.S. Mainland Heritage and Lifestyle

1) How knowledgeable is **your child** of **U.S. Mainland** culture and lifestyle?	Not at all knowledgeable	Somewhat not knowledgeable	Neutral or no response	Somewhat knowledgeable	Very Knowledgeable
2) How involved is **your child** in **U.S. Mainland** culture and lifestyle?	Not at all involved	Somewhat not involved	Neutral or no response	Somewhat involved	Very involved
3) How does **your child** feel toward the **U.S. Mainland** culture and lifestyle?	Very Negative	Somewhat negative	Neutral or no response	Somewhat positive	Very Positive

CHRS#: 21-120 | Approval Date: 10/22/2021 | Expiration Date: 10/22/2022

4) How often does **your child** associate with people of **U.S. Mainland** culture and lifestyle?	Not at all	Very little of the time	Neutral or no response	Somewhat often	Most of the time

Date: ____________ Interviewer Initials: ___________ ID#: _______________

SCREEN TIME WEEKDAY (CHILD)

	HOURS									
	0	1	2	3	4	6	7	8	9	10+
**On a usual weekday** (Monday to Friday), **how many hours a day does your child spend watching television and/or videos/DVD?**	☐	☐	☐	☐	☐	☐	☐	☐	☐	☐
**On a usual weekday** (Monday to Friday)**, how many hours a day does your child spend playing SEDENTARY video games** (DS, PlayStation, XBOX, Wii computer games, etc.)**?**	☐	☐	☐	☐	☐	☐	☐	☐	☐	☐
**On a usual weekday** (Monday to Friday), **how many hours a day does your child spend playing ACTIVE video games** (DS, PlayStation, XBOX, Wii computer games, etc.) **that incorporate movement or exercise?**	☐	☐	☐	☐	☐	☐	☐	☐	☐	☐

Which of these (television, videos/DVDs) does your child most frequently watch on a usual weekday?

____

Which of these activities (DS, Play station, XBOX, Wii computer games) does your child most frequently play on a usual weekday?

____

Which of these interactive activities (DS, Play station, XBOX, Wii computer games) does your child most frequently play on a usual weekday?

____

CHRS#: 21-120 | Approval Date: 10/22/2021 | Expiration Date: 10/22/2022

Date: ____________ Interviewer Initials: ___________ ID#: _______________

SCREEN TIME WEEKEND (CHILD)

	HOURS									
	0	1	2	3	4	6	7	8	9	10+
**On a usual weekend** (Saturday and Sunday), **how many hours a day does your child spend watching television and/or videos/DVD?**	☐	☐	☐	☐	☐	☐	☐	☐	☐	☐
**On a usual weekend** (Saturday and Sunday), **how many hours a day does your child spend playing SEDENTARY video games (DS, PlayStation, XBOX, Wii computer games, etc.)?**	☐	☐	☐	☐	☐	☐	☐	☐	☐	☐
**On a usual weekend** (Saturday and Sunday), **how many hours a day does your child spend playing ACTIVE video games** (DS, PlayStation, XBOX, Wii computer games, etc.) **that incorporate movement or exercise?**	☐	☐	☐	☐	☐	☐	☐	☐	☐	☐

Which of these (television, videos/DVDs) does your child most frequently watch on a usual Saturday and/or Sunday?

____

Which of these activities (DS, Play station, XBOX, Wii computer games) does your child most frequently play on a usual Saturday and/or Sunday?

____

Which of these interactive activities (DS, Play station, XBOX, Wii computer games) does your child most frequently play on a usual Saturday and/or Sunday?

____

CHRS#: 21-120 | Approval Date: 10/22/2021 | Expiration Date: 10/22/2022

Date: ____________ Interviewer Initials: ___________ ID#: _______________

SLEEP (CHILD)

Please complete the questions below regarding your child's sleep behavior.

1. How many hours of sleep on average does your child get in a 24-hour period (at night and in naps)? (Please choose one, ☑; h= hours)

0h	0.5h	1h	1.5h	2h	2.5h	3h	3.5h	4h	4.5h	5h	5.5h	6h	6.5h
☐	☐	☐	☐	☐	☐	☐	☐	☐	☐	☐	☐	☐	☐
7h	7.5h	8h	8.5h	9h	9.5h	10h	10.5h	11h	11.5h	12h	12.5h	13h	>13h
☐	☐	☐	☐	☐	☐	☐	☐	☐	☐	☐	☐	☐	☐

2. How long after going to bed does your child usually fall asleep?

☐ 0 to less than 15 minutes
☐ 15 to less than 30 minutes
☐ 30 to less than 45 minutes
☐ 45 to less than 60 minutes
☐ More than 60 minutes

3. Your child goes to bed reluctantly (hesitant, slowly, involuntary) ...

☐ The sleep behavior never occurs
☐ The behavior occurs once or twice a month
☐ Occurs one or two times a week
☐ Occurs between three and five nights a week
☐ The sleep behavior happens every night

4. Your child has difficulty getting to sleep at night (and may require a parent to be present)

☐ The sleep behavior never occurs
☐ The behavior occurs once or twice a month
☐ Occurs one or two times a week
☐ Occurs between three and five nights a week
☐ The sleep behavior happens every night

CHRS#: 21-120 | Approval Date: 10/22/2021 | Expiration Date: 10/22/2022

Date: ____________ Interviewer Initials: ___________ ID#: _______________

5. Your child does not fall asleep in his or her own bed

- ☐ The sleep behavior never occurs
- ☐ The behavior occurs once or twice a month
- ☐ Occurs one or two times a week
- ☐ Occurs between three and five nights a week
- ☐ The sleep behavior happens every night

6. Your child wakes up two or more times in the night

- ☐ The sleep behavior never occurs
- ☐ The behavior occurs once or twice a month
- ☐ Occurs one or two times a week
- ☐ Occurs between three and five nights a week
- ☐ The sleep behavior happens every night

7. After waking up in the night, your child has difficulty falling asleep again by himself or herself

- ☐ The sleep behavior never occurs
- ☐ The behavior occurs once or twice a month
- ☐ Occurs one or two times a week
- ☐ Occurs between three and five nights a week
- ☐ The sleep behavior happens every night

8. Your child sleeps in the parent’s bed at some time during the night

- ☐ The sleep behavior never occurs
- ☐ The behavior occurs once or twice a month
- ☐ Occurs one or two times a week
- ☐ Occurs between three and five nights a week
- ☐ The sleep behavior happens every night

9. If your child wakes, he or she uses a comforter (e.g. pacifier, binky) and requires a parent to replace it

- ☐ The sleep behavior never occurs
- ☐ The behavior occurs once or twice a month
- ☐ Occurs one or two times a week
- ☐ Occurs between three and five nights a week
- ☐ The sleep behavior happens every night

CHRS#: 21-120 | Approval Date: 10/22/2021 | Expiration Date: 10/22/2022

Date: ____________ Interviewer Initials: ___________ ID#: _______________

10. Your child wants a drink during the night (including breast or bottle-feed)

- ☐ The sleep behavior never occurs
- ☐ The behavior occurs once or twice a month
- ☐ Occurs one or two times a week
- ☐ Occurs between three and five nights a week
- ☐ The sleep behavior happens every night

11. Do you think your child has sleeping difficulties?

☐ Yes ☐ No

Please explain:

CHRS#: 21-120 | Approval Date: 10/22/2021 | Expiration Date: 10/22/2022

Date: ____________ Interviewer Initials: ___________ ID#: _______________

STRESS (CHILD)

Please respond to each question or statement by marking one box per row.

In the past 7 days…	Never	Rarely	Sometimes	Often	Always
My child felt stressed.	☐	☐	☐	☐	☐
My child felt that his/her problems kept piling up.	☐	☐	☐	☐	☐
My child felt overwhelmed.	☐	☐	☐	☐	☐
My child felt unable to manage things in his/her life.	☐	☐	☐	☐	☐
Everything bothered my child.	☐	☐	☐	☐	☐
My child felt under pressure.	☐	☐	☐	☐	☐
My child had trouble concentrating.	☐	☐	☐	☐	☐
My child felt he/she had too much going on.	☐	☐	☐	☐	☐

CHRS#: 21-120 | Approval Date: 10/22/2021 | Expiration Date: 10/22/2022

Date: ____________ Interviewer Initials: ___________ ID#: _______________

STRESS (CHILD 7+yr.)

Please respond to each question or statement by marking one box per row.

In the past 7 days...	Never	Rarely	Sometimes	Often	Always
I felt stressed.	☐	☐	☐	☐	☐
I felt that my problems kept piling up.	☐	☐	☐	☐	☐
I felt overwhelmed.	☐	☐	☐	☐	☐
I felt unable to manage things in my life.	☐	☐	☐	☐	☐
Everything bothered me.	☐	☐	☐	☐	☐
I felt under pressure.	☐	☐	☐	☐	☐
I had trouble concentrating.	☐	☐	☐	☐	☐
I felt I had too much going on.	☐	☐	☐	☐	☐

CHRS#: 21-120 | Approval Date: 10/22/2021 | Expiration Date: 10/22/2022

Date: ____________ Interviewer Initials: ___________ ID#: ______________

PARENT-CHILD INTERACTIONS (CHILD 7+yr.)

MOTHER	False<br>Really	False<br>Sort of	True<br>Sort of	True<br>Really
1. My mother exercises or plays a sport.	1	2	3	4
2. My mother and I do active things together (like walking, bike riding, playing sports)	1	2	3	4
3. When my mother does something active, she lets me do it with her.	1	2	3	4
4. My mother takes me to places where I can be active (like to team sport practices and meets, the park).	1	2	3	4
5. My mother signs me up for team sports or clubs like soccer, basketball, and dance.	1	2	3	4
6. My mother watches me play sports or do other activities like martial arts and dance.	1	2	3	4
7. My mother lets me watch TV as much as I want*	1	2	3	4
8. My mother lets me use the computer as much as I want for things that aren't homework like playing games, surfing the internet, and sending emails*	1	2	3	4
9. My mother lets me play video games (such as Play Station, Xbox, and Gameboy) as much as I want*	1	2	3	4

CHRS#: 21-120 | Approval Date: 10/22/2021 | Expiration Date: 10/22/2022

Date: ____________ Interviewer Initials: ___________ ID#: _______________

PARENT-CHILD INTERACTIONS (CHILD 7+yr.)

FATHER	False		True	
	Really	Sort of	Sort of	Really
1. My father exercises or plays a sport.	1	2	3	4
2. My father and I do active things together (like walking, bike riding, playing sports)	1	2	3	4
3. When my father does something active, he lets me do it with him.	1	2	3	4
4. My father takes me to places where I can be active (like to team sport practices and meets, the park).	1	2	3	4
5. My father signs me up for team sports or clubs like soccer, basketball, and dance.	1	2	3	4
6. My father watches me play sports or do other activities like martial arts and dance.	1	2	3	4
7. My father lets me watch TV as much as I want*	1	2	3	4
8. My father lets me use the computer as much as I want for things that aren't homework like playing games, surfing the internet, and sending emails*	1	2	3	4
9. My father lets me play video games (such as Play Station, Xbox, and Gameboy) as much as I want*	1	2	3	4

CHRS#: 21-120 | Approval Date: 10/22/2021 | Expiration Date: 10/22/2022
