# Supplementary material for: Pacific Islands Cohort on Cardiometabolic Health Study: rationale and design
Source: BMC Public Health. 2022 Jul 27;22:1428. doi: 10.1186/s12889-022-13783-9 (PMC9326143; doi:10.1186/s12889-022-13783-9)
Supplement: Supplementary file 2 — Additional file 2. [file 12889_2022_13783_MOESM2_ESM.pdf]

UNIVERSITY OF GUAM
UNIBETSEDÅT GUAHAN

OFFICE OF RESEARCH & SPONSORED PROGRAMS
Office of the Vice Provost

July 2, 2022

RE: PUBH-D-00619

Dear BMC Public Health Editors:

Thank you for the opportunity to submit our manuscript entitled "Pacific Islands Cohort on Cardiometabolic Health Study: Rationale and Design " to BMC Public Health. As requested in your July 1, 2022 email communication, we make the following responses to your inquiries:

1) The mention of Table 3 in the manuscript was an error; it should have read "Table 2". The correction has been made in the updated version of the manuscript.

2) Ethical Approval Documentation. Copies of all ethical approval from the University of Guam, College of Micronesia-FSM, and the Palau Institutional Review Board have been submitted.

3) Funding Documentation. The Pacific Islands Cohort on Cardiometabolic Health (PICCAH) Study was funded by the U.S. Department of Health and Human Services, National Institutes of Health, National Institute on Minority Health and Health Disparities (NIMHD). All official financial Notice of Award (NOA) documents have been attached to demonstrate that this study was externally funded by a manor governmental funding body. Initially this was a 5-year study, but we received supplemental funding from NIMHD in Year 4 (2019) to expand our recruitment in Palau; and then we were granted two no-cost extensions in 2021 and 2022 due to COVID-related difficulties.
   a. In addition, the PICCAH study was peer-reviewed by the NIMHD before funding was awarded in 2016, and the summary statement of that review is also included.

4) Study Status. Recruitment in Guam and Pohnpei has been completed. However, recruitment in Palau was delayed for 2 years due to the COVID pandemic shutdown of Palau. We have just been granted permission in the past few months to commence recruitment in Palau; hence, data collection has begun. Therefore, we are still in the recruitment phase of the study.

5) Related Articles. At the present time, we have presented some *preliminary* findings at the 2021 Conference of the American Society for Nutrition during a poster session. The abstract of this poster presentation can be found at: doi: 10.1093/cdn/nzab035_001. Otherwise, there are no other results published or submitted to any journal. In addition, we wish to indicate that the current manuscript nor any parts of its content are currently under consideration or published in another journal

I would like to thank you, in advance, for your time and attention. Please feel free to contact me if you need additional information or clarification on any matter regarding this submission.

Sincerely Yours,

Rachael T. Leon Guerrero

Rachael T. Leon Guerrero, Ph.D., R.D.N.
Vice Provost of Research and Sponsored Programs
Professor of Nutrition
Email: rachaeltlg@triton.uog.edu
